# The Debts We Owe

**Published:** 1884-05

**Authors:** J. A. Robinson


// THE DENTAL REGISTER.

VOL. XXXVIII.] **MAY, 1884.** [No. 5.

### COMMUNIATIONS.

### The Debts We Owe.

An address delivered at Ann Arbor, before the Alumini Association, March 25th, 1884, by J. A. ROBINSON, D.D.S.

At commencement times, at meetings like this, it is well to do as the merchants do at the beginning of the year; they take an account of their stock to see how much they have gained, and try to find out what they have lost. What is important for the man of business in a commercial point of view is equally so with the student and the professional man.

As our stock and trade of wealth does not consist entirely of merchandise or of dollars and cents, as much as what we have gained in knowledge and culture and ability to perform labor, that is actually due to our patrons; and as that labor cannot be performed by mechanical skill alone, it is proper to enquire into the source of our wealth or stock of knowledge, and see what we have on hand; see what we owe to our patrons; see what we owe to those who have gone before us, and also what we owe to those who shall come after us. The person who performs mechanical labor and leads an intellectual life may be said to live two lives in one; and if spiritually minded he has a triple life; and if length of life depends upon its usefulness, many persons have lived long whose days and years have been few. In the early days of a profession, in the new departure from the old and established customs, whatever progress is made is largely due to the persistent energy and labor of a few great souls. As all light and heat come from one central sun, so all power comes from truth; hence the great soul is only the embodiment of a great truth. A man may be an Ephod with his regalia, and occupy high positions, but without this embodiment of truth he rarely leaves anything behind him that will show that he has lived. He may amass wealth and wield it with the power of a cyclone, but without truth it soon passes away. There is a sort of self-consciousness about truth that is aggressive like the sunlight. It seems to be an *entity*—a something that is moving about in the world occupying all space without space, and all time without time. Truth and light are the same, only truth is the form of love, because we love the truth; and light is the form of beauty, as it manifests itself to man. A thing to be beautiful must be useful—a thing of utility. The beauty of a rainbow is only an incidental thing caused by the sun's rays, while the action of the elements that produce the rainbow are essential features in the uses of the atmosphere, and the rainbow is refining to our sensibilities, as is also the odor of the rose. Use is the form of beauty, as beauty is the form of truth. That use is the product of truth is seen in our constitution and laws; in all civil governments and legislation, in all the learned professions, and in all mechanical appliances. It is only when we doubt, that we hesitate, hence doubt and darkness correspond. They are the same thing called by a different name.

The account given of the great teacher in the New Testament says: "In him was light, and his light was the life of men." So it was the beauty of his life that made it the light and life and love and truth of the world. Jesus had no Alma Mater, no cherished mother, nor Lincoln, nor Garrison, nor John Brown, nor Dickens, nor Socrates; but their great souls drank in the truth and spread the light that has illumined the world. How many sit supinely down and deplore their condition because they were not endowed with genius. The greatest genius that has ever been bestowed upon man is the ability to labor in the right direction. "Omnia Vincit Labor" was the motto of the great Cæsar. Labor rightly directed rises into talent, and talent rises into genius—vigor of mind. Intellectual attainments are only the products of labor. Nothing can reproduce that is not something in itself, and that something is light and truth and life from the infinite that pervades all things, and all things are beautified by the labor of the infinite. We return each succeeding year to our cherished mother. It seems to be in the constitution of things that we rally round and reverence the fountain from which we drank the healing draught to our minds that pointed us on the way of strength and attainments. All the outlying circles of life seem to have a common center to which they are loyal. It is seen through all nature from the solar system down to the family altar. And so we grow more and more reverend as the years roll round when we contemplate the privileges we have enjoyed, and bless those who have led us by the hand up to the truth and the light. We gladly give the scepter to those who have succeeded in helping us realize our highest hopes and fondest expectations. Some one has said of Charles Dickens that he seemed to be so much above all criticism and jealousy ; he was so assured of his position ; he was so sure of the light he saw that he could write without fear or restraint the real life and truth of the world.

We look with reverence upon the great hearts that seem to have perfected in them what only seems rudimental in us. That beauty in the form of truth every true dentist can understand, for the more perfect imitations we bestow the more beautiful they become. Whenever the perfection of art conceals the artificial it is true in form and produces true happiness. In our own profession whenever the delusion escapes detection all are made happy. As all outward nature is a world of truth we are made happy in its contemplation, which shows that the human mind has a natural affection for the truth. It is by labor on the abstractions of truth that they become concretions in man ; the force that blossoms the rose is the labor of the infinite. Everything human is relatively good or bad, and it is especially so in our own profession ; hence when we come together to examine ourselves and take council to see how we stand if we have made no progress we have deteriorated, for there is no stand still in any profession. Truth is a unit, and never at variance with itself. Improvements are always brought about by the reflections of truth that has dawned in some great minds. It is through great minds that things take on forms and become practical helps or factors to the masses. The masses never bring about any particular reform. What Mathew Arnold calls the "Remnant of Society" help it to make progress, because they organize it. The numbers only disintegrate it, so that it can be molded anew. They furnish the arena for the new dispensation. The masses never follow a principle until it is embodied in some person. It is only when the principle takes possession of the masses that we see the effects of the one great mind.

The Alma Mater is the remembrance of a dear mother. It is the mother to which we look for encouragement and strength. When we turn away from here to a world of toil; when we leave this meeting we shall be filled with a sort of divineness as when receiving a blessing from a mother that will be an incentive to duty.

We find in an associated meeting like this a larger stature than our own, and the remembrance of it will give inspiration like the blessing of a mother; hence these meetings are a sort of "Mecca' that it is our duty and privilege to make a pilgrimage to every returning year. But if we owe a debt to the great souls who have helped to elevate our profession in the past, we owe a debt equally to those who are to come after us. What great soul shall lead us to greater excellence in the years that are before us? We have the personal standards of many good men to follow, and the associated wisdom of half a century on our banners. The great mistakes must be corrected; the "crooked places made straight and the rough places plain."

There are some truths that are fundamental in our profession; things that must be insisted on if we go forward and expect to retain the respect and confidence of the generation in which we live, and these things we owe to those who shall come after us. We must fight against the evils of extracting teeth and amalgam fillings. If we deceive ourselves, which seems at first thought to be of little consequence, it is the greatest calamity that can befall us. By deceiving ourselves we become cynical, and cynicism is fatal to progress. The first great untruth that prevails to an alarming extent is tooth extraction. Tooth extraction must pass away with calomel and the lancet. Even hydrargyrum cum creta is fast being pushed aside for milder forms of medicine. There are 20,000,000 of teeth extracted annually in the United States. Of this 20,000,000 fifteen millions are sacrificed to the juggernaut of nitrous oxyde, chloroform, and ether. Instead of saying the *folly* of extracting teeth, we should say the *crime* of extracting teeth. It is harder to unlearn a thing than to imbibe a wrong principle. Tooth extraction is inherited from the fathers; they never dreamed of a more excellent way. It belongs to the days of barbarism when everything that offended was destroyed. To extract good teeth because they are painful, or because it is more convenient to insert a full denture than to make a partial one is murder in the first degree. It is a capital offence against society. We repeat what we have said so many times before; the motto of the dentist should be "No teeth extracted." Treatment of diseased teeth and gums correspond to our advanced society. The cure of ulcerated teeth should keep pace with our reform schools for bad children, and must become part of our advanced civilization. This inheritance we must leave to those young in the profession; this debt we owe to those who shall come after us.

The second great debt we owe to those who shall come after us, is to rid the profession and the world of the idea that teeth can be permanently saved by filling with amalgam. I do not believe there has ever been a successful operation with an amalgam filling on an approximal surface running below the gum. Teeth are saved by oxydation (paradoxical as it may seem), and sometimes on the grinding surfaces, but never under the free margin of the gum, where the moisture of the mouth and the flap of the gum excludes the action of oxygen. We often find teeth with good sized cavities where the progress of decay is very slow; almost imperceptible for years without filling at all, and also where sections have been broken off by accident. Disintegration is very slow where the decay is very black, and this black substance found under the amalgam filling, and where the tooth has never been filled are almost identical when examined under the microscope. The oxygen, acting upon tooth substance, finds hydrogen, phosphorous, and the other ingredients more easily oxydized than the carbon. As the carbon, when not crystallized, is black or ebonized, so by oxydation of the other ingredients the carbon is set free, and is really animal charcoal and that discolors the tooth. This being antiseptic saves the tooth. It first attacks the soft tissue between the enamel rods, and finally covers or veneers the cavity or the tooth. In various conditions of the mouth chlorine is liberated from the soluble chlorides in the buccal fluids. Now, in such cases, if poorly calcified teeth have been filled with tin or the fibrous metallic filling the chlorine will combine with the tin, forming chloride of tin, which is antiseptic, and protects the organic matter from decay to some extent, and this is the reason the tin or fibrous filling is an admirable filling for the teeth of the young and for soft teeth generally. And it is the reason, as well, that the margins of the cavities round and under these fillings are not found black as when filled with amalgam. If the section of tooth is broken off and covered with moisture most of the time it is only a little brown like the leathery decay we find immediately over the pulp because the saliva of the mouth with other substances excludes the action of oxygen. In the case of certain teeth the nature of oxygen finally arrests its own progress. If it is true that teeth are saved in this way, as I have suggested, and as often, or more often, *without filling* in certain cases as when filled with amalgam, then the poor old fashioned amalgam of forty years ago, when first introduced in Boston by Dr. Mann, that was made by filing an old fashioned quarter of a dollar and mixing it with mercury was better than that now in use, because it hastened the condition I have endeavored to set forth.

I shall have to repeat here what I said in a former article, that all substance, and in particular amalgams, the process of crystallization necessarily produced shrinkage, and, of course, leaves openings between the filling and the walls of the cavity. Dr. Fletcher, of London, England, said I was mistaken, and cited water as an exception, because it caused the bursting of water pipes. At first it would seem as though his statement was correct; but if you take crystals of a snowflake you will find the crystals contract in process of crystallization. They are also thrown out of place by the frost or cold like so many needles thrown in a box promiscuously, and the changing of places is what augments the size of the mass and not the crystals themselves.

Oxyphosphates and gutta percha stoppings are far better fillings while they last, for they hermetically seal the cavity; and a metallic filling that is absolutely without recoil and can be carried to place by pressure (as wooden vessels were formerly calked to prevent their leaking) is by far the best substance that has yet been presented to the profession. Metallic substance also combined with the oxyphosphates gives strength and solidity to the filling, and makes a mixture that will not wash and one that will adhere to the walls of the tooth. While visiting an agent of dental supplies the other day I found fifteen different kinds of amalgam in his stock for sale; and, if what I have said is true, and your own experience, and the confirmation of many of our best dentists affirm it, these amalgams are demoralizing to the dentist and to the community. To the community because they are a deception and promise to save teeth when they will not; and to the dentist because he is a party to the deception, and it is only for the love of ease and money that he does what he does. We cannot expect any reform from the dental depots, for they treat all goods as merchandise. The gold manufacturers will not assist in any reform that diminishes the sale of gold. They manufacture and sell to make money. We cannot expect help from the *lazy* dentist, for laziness forces him to the use of the trowel to plaster up the teeth. As we grow bad little by little, so we must plod our weary way out of these difficulties little by little. There is but one way out of this, and that is to educate the people. Their attention has been drawn in the direction of their teeth, and now they must be taught the crime of extracting teeth, and the folly of trying to save them by filling with amalgam. If the people are well informed the charlatan will be unemployed. I believe it to be the *right* and the *duty* to sow broadcast the truth through any medium to reach the public ear. In plain language, I believe in advertising, but in only such a way as will lead the community in the way of truth. If dignity of character be preserved and go before all ways of communication are open. This can be done without forgetting we belong to a profession. If dignity be preserved there will follow a magnificent treasure of result. The world has never been converted or educated by silence. The mind of the world is discarding, or rather outgrowing, the old order of things, and professions must keep pace with the telephone, the telegraph, and the railroad. We must look at things from the standpoint of friendship to the world as well as to ourselves. The narrow channel through which all the professions have worked, like the channel to the North Pole, is through a sea of snow and ice. The millions must be taught the truth in regard to their teeth, and nothing will reach them but the printing press, in their solemn but rapid march of progress and civilization. Whoever repudiates or overcomes an error is approximating a new truth. When we first discover a new truth it is only the answering of a silent question that we have never had quite the intelligence to ask. Absolute truth is generative; it pushes itself into the mind; it seems to be a thing the infinite has laid in our path that we have had the good fortune to find. When we are shown truth by others, it is mechanical; it does not come from the soul outward. All discovered truth and light come from within, and when they are stimulated by outward nature they take form and grow. Thought is a spiritual force within us, which being brought into contact with outward nature produces action, and manifests itself by re-action, as all seeds grow. When we have found a truth or have a new thought we only become great as we give it away. When we have given it away it becomes more our own, because in giving we enlarge our capabilities. Amalgam is the tool of the sluggard. The sluggard commences life with a deception and a lie, and ends his life in disappointment. He deterioates while working amalgam because it does not require thought, and without thought we cannot make progress. We cannot create, we can only help preserve what is going to decay. If we cannot create we have no right to ruthlessly destroy. A professional life is only putting to the best use the highest gifts of God. The debt we owe to those who shall come after us, is to take dentistry out of the hands of the profligate tooth pullers and breathe into it a moral sweetness that will make it honorable, and give it a more elevated place among the professions.

The uses and values of life are the gifts of God, and man's organism is one of them. He has given us the light for growth and thought, and darkness for rest, and so he turned the world on its axis. By and by man took the tools of nature—the water, the wood, and the fire—and made boats and steam vessels, and sailed round the world; but he learned the use of the sail from the tail of the squirrel; the rudder from the fins of a fish, and the motion from the wings of the eagle. Then science opened to his sight another great book of Nature, and the occult relation between plant life and animal life is discovered; he read that their union and life-giving natures exactly correspond. The desire to put forth flowers and to reproduce animal life begins in the spring time. As the new covering comes to vegetable life so the soft new coat to the animals; and the fructifying desires run through bird and beast and grass and flowers and fish, and return with the annual circuit of the earth round the sun. By reaching a little farther, and with the aid of the telescope, we find the nebulosity grows into nebula, the nebula into stars, and stars into planets, and the planets into a solar systems, and so we are forced to believe that there are anthems that have never been heard by the human ear, and sights that have never been seen by the human eye, and that all the universe is one grand vibration; and, like the universal law of gravitation, tends to one universal whole, whose mind fills all space, whose personality is infinite, and whose attributes must be universal wisdom and universal love.

With this vision before us let us go forward, and as true artists try and see beyond the attainments of to-day. The painters will find flowers and clouds in their pathway; the mechanic will find waterfalls and machinery to carry his mill; the poor dentist will find nothing above tooth pulling and amalgam fillings, while the good dentist sees deformities regulated in the same manner as the horticulturist corrects deformities in his trees, and takes his ideal by studying the perfect trees; and we all find that which will satisfying the culture we have attained the natural cravings of the human heart, and in proportion, as that heart has advanced in mechanical, moral, and spiritual culture. The educated dentist sees all the difficult cases of regulation and filling; all the chronic cases of alveolar abscess and diseases of the mouth; all his prosthetic operations; the artistic arrangement of the teeth; their shade and color, hours before his patient rings at his door or announces his presence. The dentist is the product of this nineteenth century, and has grown out of our advanced civilization. To be absolutely great he must be a man of just, moral, and mechanical perceptions, and be able to demonstrate what he sees. I use the word moral because dental work demands just and conscientious labor above mere common mechanism, as the health of the patient depends on the thoroughness and success of all his operations. To do this we must commiserate our ignorance and work. We must commiserate our errors and overcome them. The dentist is an artist. He is a painter, with more than a brush and a pencil; a mechanic, with more than a saw and a hammer; a physician, with more than the M.D., and a dentist, with more than the D.D.S. He is the scholar, with the past and the present within him; the man of truth and heart to help the world onward toward perfection. This man has grown out of the freedom of our institutions. The freedom of the eye, the hand, and the heart, for dentistry must have utility, strength, and beauty. They imply intellect, morals and force, strength of muscle, and facility, and heroism. These things, with a strenuous life, can withstand all the swing and rebound of the world. These things we owe to those who shall come after us. And where shall we find the great souls who will bear them down to coming generations?

